# Extracellular signals regulate the biogenesis of extracellular vesicles

**DOI:** 10.1186/s40659-022-00405-2

**Published:** 2022-11-26

**Authors:** Yong Jin, Lele Ma, Wanying Zhang, Wen Yang, Qiyu Feng, Hongyang Wang

**Affiliations:** 1 Cancer Research Center, The First Affiliated Hospital of USTC, Division of Life Sciences and Medicine, University of Science and Technology of China, Hefei, 230036 Anhui People’s Republic of China; 2 National Center for Liver Cancer, Eastern Hepatobiliary Surgery Hospital/Institute, The Second Military Medical University, Shanghai, 20815 China

**Keywords:** Extracellular vesicle, Exosome, Microvesicle, Extracellular signal, Signal transduction, Information transfer

## Abstract

Extracellular vesicles (EVs) are naturally released membrane vesicles that act as carriers of proteins and RNAs for intercellular communication. With various biomolecules and specific ligands, EV has represented a novel form of information transfer, which possesses extremely outstanding efficiency and specificity compared to the classical signal transduction. In addition, EV has extended the concept of signal transduction to intercellular aspect by working as the collection of extracellular information. Therefore, the functions of EVs have been extensively characterized and EVs exhibit an exciting prospect for clinical applications. However, the biogenesis of EVs and, in particular, the regulation of this process by extracellular signals, which are essential to conduct further studies and support optimal utility, remain unclear. Here, we review the current understanding of the biogenesis of EVs, focus on the regulation of this process by extracellular signals and discuss their therapeutic value.

## Introduction

**Fig. 1:**
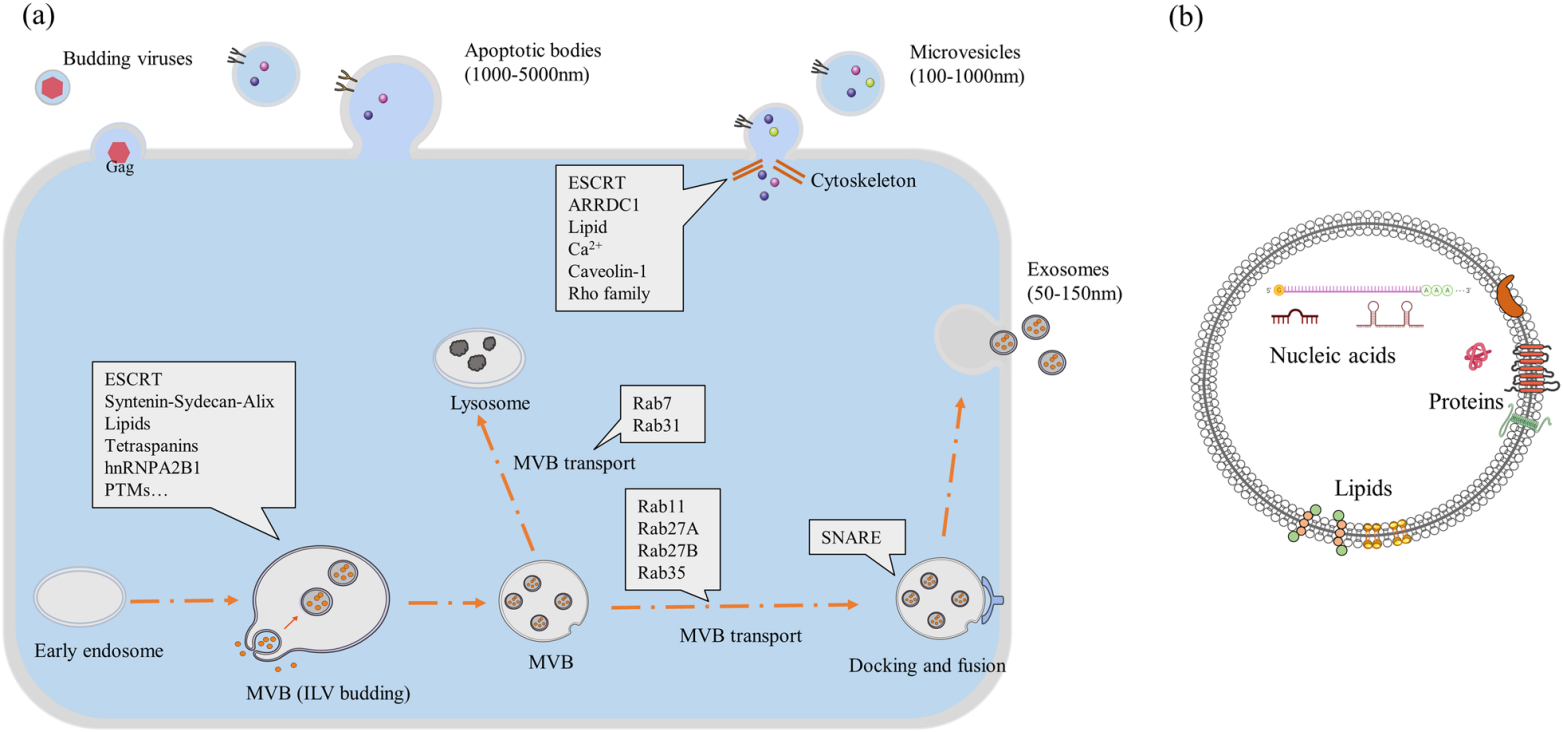
The biogenesis of the extracellular vesicle and its structure. **a** EVs can be divided into three subtypes: exosomes, microvesicles and apoptotic bodies. Exosomes are formed as ILVs in the MVBs. This process requires the involvement of ESCRT components, and it also occurs in ESCRT-independent pathways, including syntenin-, lipids- and tetraspanin-dependent mechanisms and others. After ILVs formation, MVBs are transported to the plasma membrane or the lysosome, which primarily involves some Rab proteins. Finally, MVBs fuse with the plasma membrane with the involvement of SNARE complex, and exosomes are released. Microvesicles are released directly after the outward budding from the plasma membrane, which primarily involves the ESCRT, ARRDC1, lipids, Rho proteins and Ca^2+^. Apoptotic bodies only generate from apoptotic cells and shed from the cell surface. Enveloped viruses that highjack the membranes for release can also be considered as a kind of EV. **b** The extracellular vesicle is made up of lipid bilayers and enriched in proteins, nucleic acids and lipids

Extracellular vesicles (EVs) are lipid bilayer membrane structures that are released by almost all types of cells during normal physiology and acquired abnormalities [1, 2]. Based on their biogenesis and size, EVs are commonly classified into three subtypes: exosomes, microvesicles (MVs), and apoptotic bodies. Exosomes, 50–150 nm in diameter, are intraluminal vesicles (ILVs) formed by the inward budding of the endosomal membrane during the maturation of multivesicular bodies (MVBs), which are secreted after the fusion of MVBs with the plasma membrane [3, 4]. MVs, about 100 to 1000 nm in diameter, are formed by the outward budding of the plasma membrane and are released directly into the extracellular milieu [5]. Apoptotic bodies are generally defined as 1 to 5 μm in diameter and as products of apoptotic cell disassembly [6]. As shown in Fig. 1, all these three subtypes are composed of lipid bilayers and enriched in proteins, lipids, and nucleic acids (Fig. 1) [7, 8].

Although initially considered as a form of discarding waste of cells and “platelet dust”, exosomes and MVs are now extensively characterized [9–12]. This is because they are central mediators of cell-to-cell communication and are involved in almost all physiological and pathological processes [13]. For instance, EVs play critical roles in inflammation, stem cell expansion, and diseases including cancers and neurodegenerative disorders [9, 14–17]. In recent years, research on the applications of EVs in diagnoses and treatments of various diseases has been increasing. EVs are present in all body fluids, facilitating easy clinical sampling and have the potential for longitudinal sampling to track disease progression [2]. In addition, exosomes are being actively explored as drug delivery vehicles due to their small size, endogenous, less toxic, etc. [18].

Studies on the functions of EVs have attracted a lot of attention and been quite extensive over the past decade. However, relatively few studies have been conducted on the biogenesis of EVs, especially on how extracellular signals regulate the biogenesis of EVs. Compared to the classical signal transduction that works in diffusible mono-molecule form, EVs demonstrate remarkable efficiency and specificity through assembling their various and selective cargo, providing novel mechanisms of signal transfer. More importantly, the altered biogenesis of EVs reflect changes in the signaling or metabolic state of donor cells, making them versatile. Therefore, it is necessary to elucidate the regulatory mechanisms of EV biogenesis before EVs are put into application. Here, we review the development of EV research and current understanding of the biogenesis of EVs, emphasize the regulation of this process by extracellular signals, and discuss their potential therapeutic value.

## The development of extracellular vesicle research

Extracellular vesicles were first isolated from platelets by high-speed centrifugation and were reported to inhibit plasma clotting in 1946 [19]. These vesicles are actually microvesicles as now defined. Thirty-seven years later, Harding et al. and Pan et al. reported that transferrin receptors associated with small vesicles are released from reticulocytes into extracellular space by two groups, respectively [20, 21]. Unexpectedly, their research on the mechanisms of transferrin recycling in reticulocytes raised the possibility that cells might use MVB exocytosis as a general way to release vesicles [22]. Since then, many proteins, such as class II major histocompatibility complex, were demonstrated to be transported in MVB-exosome pathway and to play biological functions, and the understanding of the cell biology of exosome increased [22, 23]. In 2007, it was firstly reported that exosomes contain both mRNA and microRNA, which can be delivered to another cell and exerted their functions [24]. This groundbreaking discovery introduced EVs into intercellular communication and opened up a new field of research with widespread interest.

In recent years, the research on EVs has been gradually standardized under the guide from the International Society for Extracellular Vesicles (ISEV), including nomenclature, isolation method, characterization, and functional studying method. It is now universally acknowledged that exosomes are endosome-derived vesicles released from cells, while microvesicles are plasma membrane-derived, although specific markers of EV subtypes are not clear enough and it is hard to trace their origin in experiments [25, 26]. The separation and concentration of EV, the key step in the experiment, was summarized in the guideline of ISEV [26]. The common methods include differential ultracentrifugation, density gradients, precipitation, filtration, size exclusion chromatography, and immunoisolation [26]. In addition, additional techniques and combinations of techniques were applied to get better performance, such as asymmetric flow field-flow fractionation, ion exchange chromatography [27, 28]. Another important step is EV characterization, some methods are recommended to assess the quantification and purity of EVs, such as using light scattering technologies to count particle number, using electron microscopy to visualize EVs, and selecting EV protein compositions (CD63, CD9, CD81, TSG-101, ALIX, etc.) or non-protein components (glycosphingolipid, ceramide, etc.) as markers [26]. Given the fact that there is no ideal technology to distinguish EV subtype from each other, strict and clear definition of EV and comprehensive methods are needed.

## The biogenesis and release of extracellular vesicles

### The biogenesis of exosomes

As shown in Fig. 1, during the maturation of early endosomes into late endosomes or MVBs, endosomal membrane loaded with specific cargo buds inward and separate from the membrane, generating ILVs for exocytosis within the lumen of the endosomes (Fig. 1) [29]. In general, the mechanisms of exosomes biogenesis can be divided into the Endosomal Sorting Complex Required for Transport (ESCRT)-dependent pathway and ESCRT-independent pathways.

#### ESCRT

ESCRT consists of about thirty proteins that can be divided into four complexes, ESCRT-0 and ESCRT-I, II, III [30–34], and the discovery of them reveals the cellular mechanism underlying the biogenesis of exosomes [35, 36]. The formation of ILVs begins with cargo loading on the limiting membrane of MVBs, which is mediated by a multivalent ubiquitin-binding complex ESCRT-0 composed of hepatocyte growth factor-regulated tyrosine kinase substrate (HRS) and signal transducing adaptor molecule (STAM) [37, 38]. ESCRT-0 contains ten ubiquitin-binding sites that facilitate capturing polyubiquitylated cargo [38]. When the ubiquitinated cargo binds to the ESCRT-0 complex, HRS-STAM complex recruits ESCRT-I complex to transmit cargoes through TSG-101 binding ubiquitin [38]. Subsequently, ESCRT-I recruits ESCRT-II through the connect of VPS28 and VPS36 subunit [39]. Finally, ESCRT-III is recruited by ESCRT-II to nucleate charged MVB protein 2-4 (CHMP2-4) polymers to completes ILVs budding and fission of this membrane [39]. In addition, the accessory protein AAA-ATPase VPS4 disassembles and recycles ESCRT-III [8].

#### Syntenin

There is an alternative pathway playing key roles in the biogenesis of exosomes, which involves in syntenin, syndecan, and ESCRT accessory protein ALG-2 interacting protein X (ALIX) [40]. Syntenin binds to the cytoplasmic domain of syndecan and recruits ALIX, which allows inward budding into ILVs together with ESCRT-I, II, III components. This process relies on Src-mediated endocytosis of syndecan-1 and requires PLD2 and ARF6 GTPase activities [41–43]. Recently, it was reported that Src homology 2-containing protein tyrosine phosphatase 2 (Shp2) controls exosome biogenesis through dephosphorylation of syntenin, thereby regulating exosomes-mediated epithelial-macrophage crosstalk [44].

#### Lipids

Exosome biogenesis also occurs in the absence of ESCRTs, and several studies have shown that MVBs still form despite maximal inhibition of ESCRT-dependent pathway [45]. Trajkovic et al. revealed the ESCRT-independent pathway for exosome biogenesis and showed that the transfer of exosome-associated domains into the lumen of the endosome depends on the sphingolipid ceramide in the mouse oligodendroglial cells [46]. Ceramide can induce small microdomains to coalesce into larger domains, thereby facilitating domain-induced budding [47]. Moreover, the tapering structure of ceramides lead to spontaneous negative curvature by creating area differences between the membrane leaflets [48]. Cholesterol, an important component of MVBs, is another lipid enriched in exosomes membranes [49–51]. It was demonstrated that induced cholesterol accumulation in late endosomal compartments of the oligodendroglial cells increases the secretion of exosomes in a flotillin-dependent way [52].

#### Tetraspanins

In addition to lipids, proteins from tetraspanin family are involved in the regulation of cargo sorted for exosome secretion. This family is characterized by four transmembrane domains and has as many as thirty-three members in mammals, including CD9, CD37, CD51, CD53, CD63, CD81, CD82, etc. [53–55]. The first study showed the tetraspanin-dependent mechanism of cargo sorted to ILVs, focusing on CD63, which is involved in endosomal sorting in melanoma cells [56]. Subsequent studies demonstrated that CD63 was also involved in cargo targeting exosomes secreted by melanoma cells and in the biogenesis of exosomes in fibroblasts from patients with Down syndrome [57, 58]. CD81 is also a member of the tetraspanin family and enriched in exosomes and was shown to target its ligands array into secreted exosomes in mouse lymphoblasts [59]. Expression of the CD9 and CD82 was reported to augment the exosome release of β-catenin from HEK293 cells [60]. Interestingly, tetraspanin Tspan8 is involved in exosome biogenesis in rat adenocarcinoma cells by contributing to selective recruitment of proteins and mRNA into exosomes without affecting the amount of secreted exosomes [61, 62].

#### Other mechanisms involved in the exosome biogenesis

There are other mechanisms involved in the biogenesis of MVBs. For example, soluble proteins isolated into ILVs rely on the chaperones HSC70 which recruits the transferrin receptor (TFR) to exosomes and bind cytosolic proteins containing a KFERQ-motif to be selectively transferred to ILVs in reticulocytes [13, 63, 64]. Exosomes also carry nucleic acids including DNA sequences and RNAs [24, 65–67]. The Goldie group demonstrated that in small RNAs, the proportion of miRNAs in exosomes is higher than that of their donor cells [68]. Intriguingly, miRNAs are not randomly loaded into exosomes [69]. miRNA can be transferred into exosomes via the SUMOylated heterogeneous nuclear ribonucleoprotein A2B1 (hnRNPA2B1), the neural sphingomyelinase 2 (nSMase2) -dependent pathway, the 3’-end of the miRNA sequence-dependent pathway, and the miRNA induced silencing complex (miRISC) -related pathway [70–73]. It has also been proposed that the RNA packed into extracellular vesicles can also be facilitated by retroviral coat proteins such as Gag (and their silent copies present in animal genomes) in *Drosophila*. These proteins effectively target the RNA they recognize to the plasma membrane or MVB membrane, resulting in the release of virus-like particles [74, 75]. In addition, post-translational modifications (PTMs) can direct cargo into exosomes. Except that ubiquitin domain of ubiquitinated proteins can be recognized by ESCRT protein TSG101, SUMOylation, ISGylation, phosphorylation, glycosylation, and acetylation of proteins can also lead to their sorting into exosomes [76, 77]. For example, ISGylation of TSG101 induces its aggregation and degradation, impairing exosome secretion [78]. SUMOylation of α-synuclein, the major protein of pathological aggregates of Parkinson’s Disease, can be sorted into MVB via the interaction between ESCRT and phosphoinositols [79].

In conclusion, the process of exosome biogenesis is complex and depends mainly on the cargo that affect the function of these exosomes. It is noteworthy that different mechanisms work simultaneously within a single cell, and therefore various subtypes of MVBs exist within a single cell. Precisely, during the maturation of MVBs, various mechanisms act simultaneously or sequentially to help release various exosomes [13].

### The release of exosomes

#### Mechanisms involved in MVB transport

After formation of ILVs, MVBs are either transported to the plasma membrane for the subsequent release of ILVs (exosomes) into the extracellular environment or are degraded by fusing with lysosomes (Fig. 1). There is a balance between these two opposing events, and some studies suggested that this balance shifts towards exosome release in transformed cells and non-transformed cells [80–82]. The underlying mechanisms of this balance are largely unknown, but some have begun to emerge, such as ISGylation of the ESCRT-I component TSG101, which impair exosome release by promoting MVBs fusion with lysosomes in mice [78].

The mobilization of MVBs to the plasma membrane is similar to the general intracellular vesicles trafficking and involves the cytoskeleton, associated molecular motors, and the molecular switches [13, 83, 84]. Rab proteins, a subfamily of the Ras superfamily of small GTPase, are involved in different steps of intracellular vesicular trafficking, from the budding and scission of vesicles from their donor membranes, to transporting of vesicles along cytoskeletal components, and then to docking of vesicles to their target compartment[85, 86]. Rabs work between an active GTP-bound state and an inactive GDP-bound state, and the switch requires guanine nucleotide exchange factor (GEF) and GTPase activating protein (GAP) [87]. Specifically, Rabs binding to the GDP dissociation inhibitor (GDI) exist in cytosol in an inactive state, while prenylated Rabs are inserted into the membrane of their respective transport compartment. Then GDP is replaced by GTP by GEFs to form an active state [87, 88]. Active Rabs can interact with various effector proteins, such as motor proteins involved in transport and tethering factors, then Rabs are inactivated by GAPs and enter to next cycle [86]. For example, Rab7 interacts with Rab-interacting lysosomal protein to recruit the dynein-dynactin motor complex to regulate the transport of late endosomes to lysosomes [86]. Savina et al. reported that Rab11 is involved in exosome release, which reduced the release of TfR and HSC70-containing exosomes in K562 cells when inhibited [89]. With further studies, several Rab proteins, including Rab27A/B, Rab7, Rab31 and Rab35, have been implicated in the regulation of exosome secretion. Rab35 was revealed to be necessary for PLP-bearing exosome release and allow docking of MVBs to the plasma membrane in oligodendroglial cells [90]. Rab7 was shown to be required for syntenin- and Alix-containing exosome secretion in MCF7 cells but not to affect exosome secretion from HeLa cells [40, 91]. Recently, Rab7 was reported to be bound and recruited to MVBs by neddylated Coro1a, promoting lysosomal degradation of the MVBs and reducing exosome secretion consequently in HEK293, RAW264.7 and HeLa cells [92]. Two isoforms of Rab27, Rab27A, and Rab27B, play a role in the secretion of exosomes bearing CD63, CD81, and MHC class II in HeLa cells and docking to the plasma membrane [91]. However, depletion of Rab27A has no effect in decreasing secretion of exosomes in certain cell lines such as MDA-MB-231 [8, 93, 94]. Recent work identified that Rab31 marks and controls an ESCRT- independent exosome biogenesis pathway and prevents the fusion of MVBs with lysosomes in HeLa cells [95]. Interestingly, Rab31 functions as a driver for ILVs formation and determines the fates of endocytic membrane proteins by balancing with Rab7 [95].

#### Mechanisms involved in MVB fusion with plasma membrane

Exosomes are released into the extracellular environment upon fusion of MVBs with the plasma membrane (Fig. 1). This process involves SNARE (soluble NSF-attachment protein receptor) proteins or complexes that allow fusion of the lipid bilayers of two different intracellular compartments [96]. VAMP7 (vesicle-associated membrane protein 7) on the lysosomes, syntaxin 7 on the plasma membrane, and the lysosomal regulatory synaptotagmin7 form a complex participating in the exocytosis of conventional lysosomes, which has been reported to be involved in the exosome secretion of K562 cells [97, 98]. Another SNARE protein YKT6, involved in endoplasmic reticulum-to-Golgi complex transport, has been shown to be required for exosome release by depletion in HEK293 cells and A549 cells [99, 100]. Moreover, syntaxin 5 and Ral 1 in C.*elegans*, syntaxin 1a in mammals and SNAP-23 in human mastocytes are regulators of the exosomal secretion process as well [81, 101, 102]. Similarly, different SNARE complexes may be applied to the fusion of a particular organelle with the plasma membrane in different cell type or the fusion of different subpopulations of MVBs in a single cell type [8].

As depicted in Fig. 1, MVBs are transported to the plasma membrane, and this process primarily involves the Rab proteins. MVBs fusion with the plasma membrane is similar to the general fusion process of membranes, which requires the involvement of SNARE complex. Notably, the above studies suggest that the mechanisms of exosome secretion may be limited by the original subpopulations of endosomes or dependent on the cell type [13, 103].

### The biogenesis and release of microvesicles

Unlike the fusion of MVBs with the plasma membrane, as illustrated in Fig. 1, the shedding of MVs requires a fission process that occurs in the curved region of the plasma membrane (Fig. 1). Mechanisms similar to exosome biogenesis are involved in the generation of MVs. ESCRT factors are involved in this process requiring ESCRT-I, II, III, and accessory proteins [42]. Among them, the arrestin domain-containing protein 1 (ARRDC1) directly drives plasma membrane budding by recruiting TSG101 to the plasma membrane, while VPS4 is also involved [38, 104]. Additionally, acid sphingomyelinase induces the production of ceramide-dependent MVs of astrocytes [105], while neutral sphingomyelinase in MVBs is required for the biogenesis of exosomes [46]. Another lipid-dependent mechanism is associated with cholesterol which is abundant in MVs, and the formation of MVs is impaired when cholesterol is pharmacologically depleted in activated neutrophils [106]. PTMs are also reported to participate in the MV biogenesis [77]. As a typical example, Y14-phophorylation of caveolin-1 leads to interaction with hnRNPA2B1, followed by induction of hnRNPA2B1 O-GlcNacylation, which triggers the binding between selected miRNAs and hnRNPA2B1, finally leading to selection of miRNAs into MVs [107].

Given the pattern and site of MVs biogenesis, cytoskeleton rearrangement and related molecules are critical to this process. Large increase in Ca^2+^ levels induces the activation of calpain, a protease capable of cleaving cytoskeletal proteins, together with the alteration of flippase, floppase, and scramblase, drives membrane asymmetry cytoskeletal remodeling, leading to outward budding of microvesicles [108–111]. Consistently, the Rho family small G protein and Rho-associated protein kinase (ROCK) are required for MVs release [112, 113]. Moreover, a recent study by Wang and Zhuang showed that Rho family small G protein Cdc42 is a convergent node of multiple regulatory signals that occur in MVs biogenesis and the binding of activated GTP-bound Cdc42 and its downstream effector, Ras GTPase-activating-like protein 1 (IQGAP1), is required for MVs shedding in breast cancer cells [113]. Also, the activation of RhoA by ARF6 or ARF1 leads to an actin-myosin-based contraction that is required for MVs budding through the kinases ROCK and ERK (extracellular signal regulated kinase) [114, 115]. Diaphanous-related formin-3 (DRF3), a signaling protein binding small Rho family GTPases, has been reported to be associated with the formation of MVs when suppressed in prostate cancer cells [116]. Furthermore, the biogenesis of MVs in cancer cells is also associated with metabolic changes driven by the “Warburg effect”, which is thought to be required for malignant transformation and cancer progression [117].

## Extracellular signals regulation of the biogenesis and release of extracellular vesicles

In cells, signal transduction networks receive and transmit signals, including those from the extracellular environment, to regulate and coordinate core cellular functions, such as the release of EVs [118]. Although several pathways for the biogenesis and release of EVs and the key regulatory molecules involved have been described and characterized, extracellular signals and associated regulatory pathways have been rarely mentioned. Indeed, the extracellular signals, receptors, and downstream molecules are intertwined with the biogenesis and release mechanisms of EVs.

### G protein-coupled receptor regulates EV biogenesis and release

**Fig. 2:**
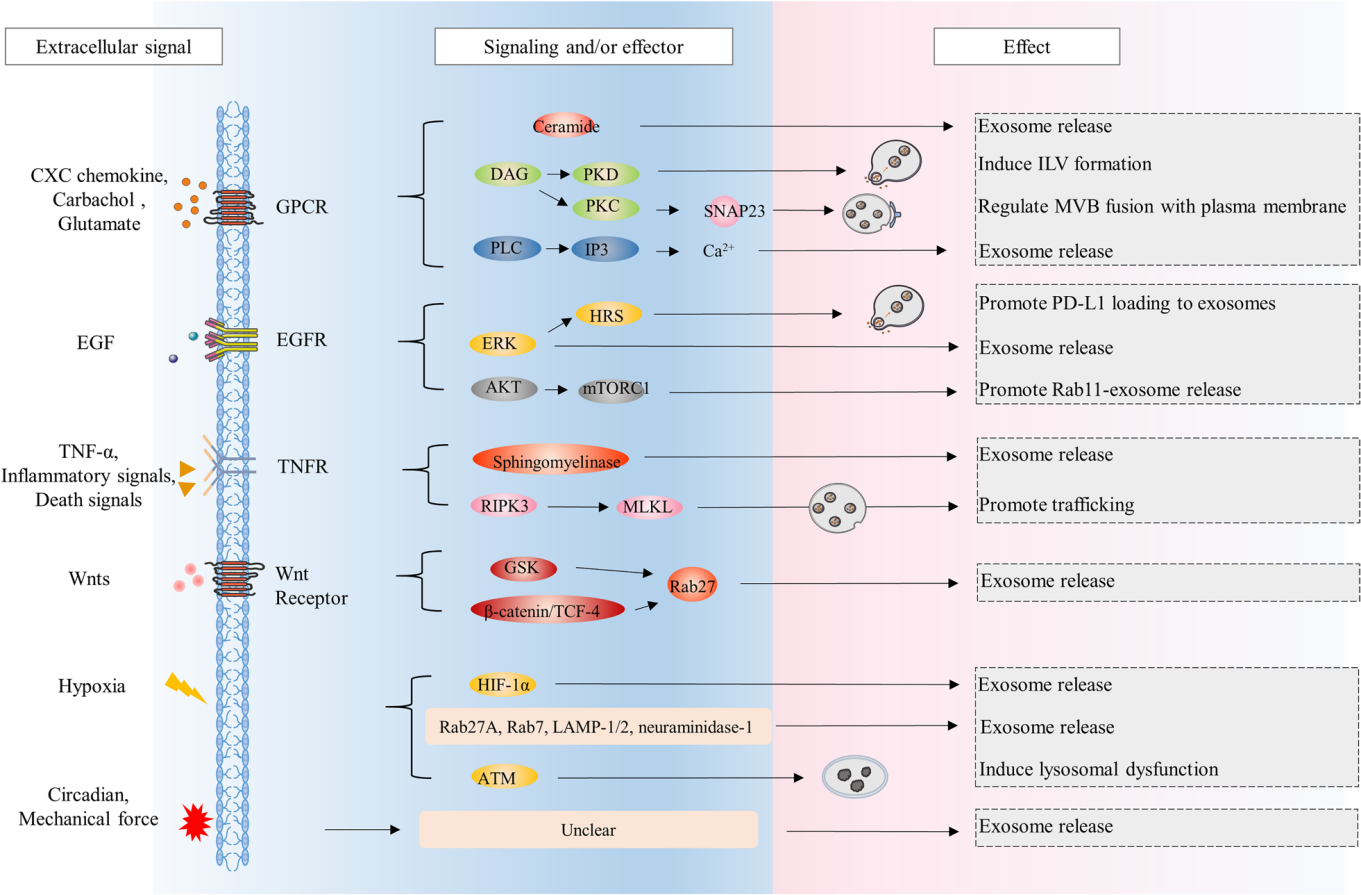
Extracellular signals regulate the biogenesis of exosomes. Extracellular signals regulate different events during the biogenesis of exosomes in different cell types. Activated GPCR regulates neutral sphingomyelinase activity and ceramide to effect exosome release, induces the formation of ILVs via DAG/PKD, triggers exosome release via PLC/IP3/Ca^2+^ and participates in MVBs fusion with the plasma membrane via DAG/PKC. Activated EGFR activate ERK and HRS to promote cargo loading and exosome release. Inhibitory AKT/mTORC1 signals delivered by EGFR stimulate release from Rab11a compartments of exosomes. TNF-α triggers the release of exosomes which depends on sphingomyelinase. Cell death inducer stimulate TNFR, then TNFR promotes endosomal trafficking via RIPK3/MLKL pathway and enhances exosomes release. Wnt-mediated GSK3 inactivation regulates the expression of Rab27, and Wnt/β-catenin/TCF-4 activates the expression of Rab27B, therefore participating in the regulation of exosomes biogenesis. Hypoxia regulates exosome release through HIF-1α, Rab27A, Rab7, LAMP-1/2, neuraminidase-1 and ATM. Circadian clock and mechanical force also regulate exosome release, but specific mechanisms are unclear

**Fig. 3:**
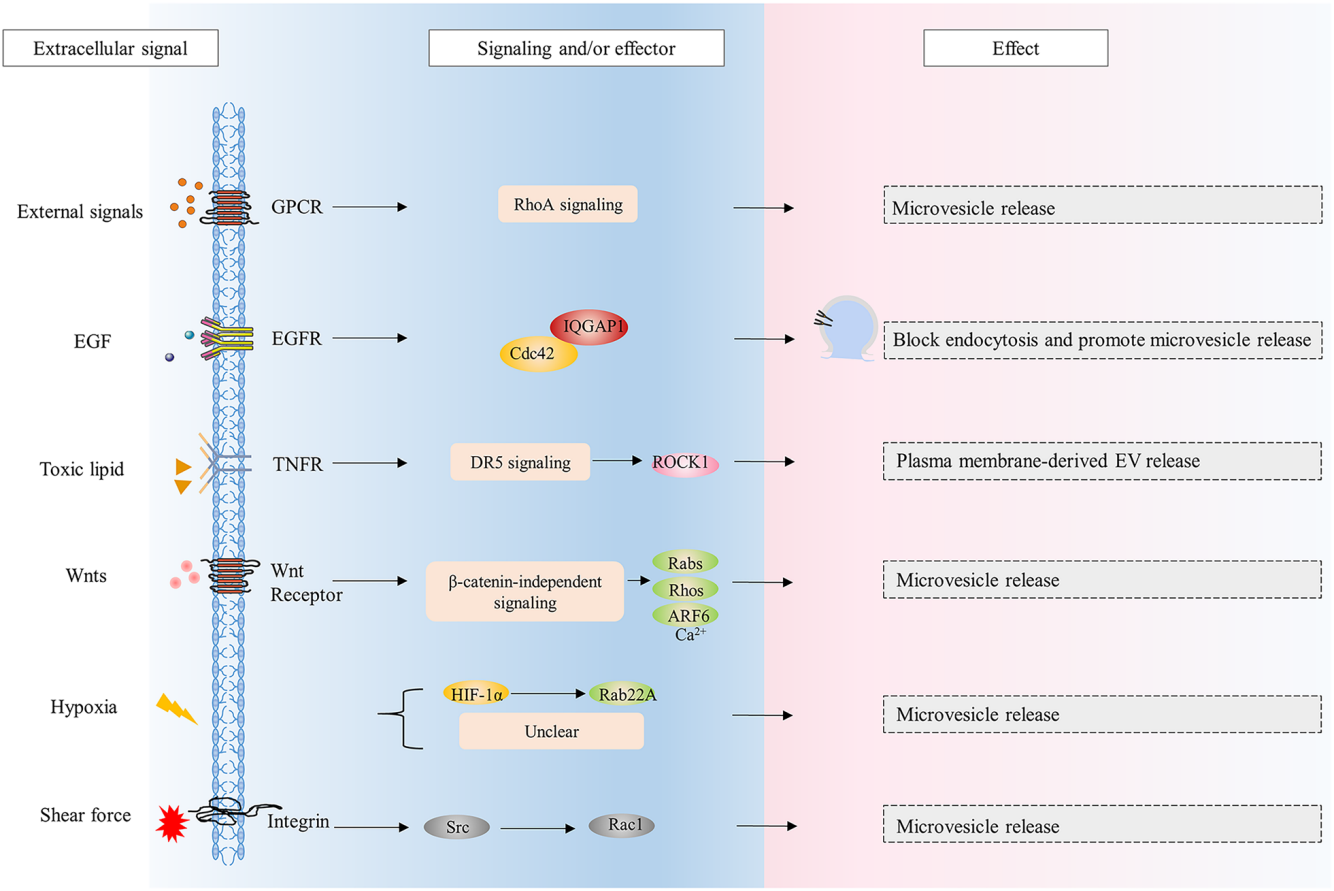
Extracellular signals regulate the biogenesis of microvesicles. Microvesicles release directly from the plasma membrane and Rho signaling is one of the most important pathways mediating GPCRs signals regulation of microvesicle release. EGF activates EGFR to activate Cdc42, activated Cdc42 binds to IQGAP1 to block endocytosis and facilitate microvesicle release. Toxic lipid through TNFR activates DR5 proapoptotic signaling and ROCK1 to regulate plasma membrane-derived EV release. Wnt signaling regulates the expression of the Rab, Rho, ARF and Ca^2+^ to affect microvesicle release. Hypoxia regulates Rab22A expression through HIF-1α to affect microvesicle release. Shear force induces microvesicle release through integrin signaling and Scr/Rac1 signaling

G protein-coupled receptors (GPCRs) belong to a superfamily of cell surface signaling proteins that play a variety of physiological functions and roles in a wide range of diseases [119]. External stimuli, such as proteins, peptides, sugars, lipids, and neurotransmitters, can activate GPCRs, leading to the activation of subsequent effectors [120]. Previous studies have shown that many GPCR signaling components are involved in EV biogenesis and release from different cell types [121–125]. For instance, CXC chemokine was demonstrated to regulate hepatocyte exosome release via CXCR2 which modulates neutral sphingomyelinase activity and resultant production of ceramide [126]. Carbachol stimulates the muscarinic M1 receptor, thereby inducing the formation of ILVs in a DAG/PKD-dependent way in T lymphocytes [127, 128]. Extracellular glutamate activates the metabotropic glutamate G-protein-coupled receptor mGluR3 to promote Rab27/CD63-dependent and mitochondrial DNA-containing EV release in breast cancer cells [129]. Moreover, Ca^2+^ functions as both the second messenger and the key regulator of the release of EVs, mediating external signals regulation of EVs release as expected. External signals combine with GPCR and increase cytoplasmic Ca^2+^ concentration by PLC/IP3 pathway, which triggers exosome release in microglia cells (Fig. 2) [130]. Meanwhile, there are some cases that the activation of multiple GPCRs stimulates EV release without raising intracellular calcium [131]. Another downstream of GPCR/PLC, Diacylglycerol/Protein Kinase C (DAG/PKC) pathway, was proved to be involved in MVBs fusion with the plasma membrane via phosphorylation of serine residue 110 of the SNAP23 in HeLa cells (Fig. 2) [132]. And beyond that, the pool of DAG in the Golgi determines the forming efficiency of post-trans-Golgi network secretory vesicle, and a negative regulator of DAG, diacylglycerol kinase alpha, was found to regulate secretion of bioactive exosomes in T lymphocytes [133, 134]. In addition to above mechanisms, external signals also can regulate the release of microvesicles via RhoA signaling as reported (Fig. 3) [114].

### EGFR regulates EV biogenesis and release

Epidermal growth factor receptor (EGFR) belongs to the ErbB family of receptor tyrosine kinases (RTKs), which plays a pivotal role in cell physiology [135]. Extracellular EGF stimulation causes receptor transphosphorylation and consequent activation of the intracellular signaling cascade, including Ras-ERK, PI3K-Akt, PLC-PKC, which ultimately results in the alteration of cell proliferation, survival, and differentiation [135, 136]. Zhou et al. studied the correlation between exosome secretion and EGFR activation in renal tubular cells, suggesting a negative regulation of EGFR signaling in exosome secretion [137]. Consistently, it was reported that inhibition of EGFR signaling triggers a burst of release of exosomes containing EGFR in human epidermal cells [138]. However, Koistinen et al. found that epithelial to mesenchymal transition induced by EGF enhances the production of CD44 and release of EVs, and proposed CD44 as a potential marker of EVs in rat primary mesothelial cells [139]. Guan et al. reported that extracellular signal-regulated kinase (ERK) mediated phosphorylation of HRS selectively promotes PD-L1 loading to exosomes through ubiquitin-independent binding and increases exosome release in metastatic melanoma cell line [140]. Kim et al. demonstrated that heparin-binding EGF activates EGFR and ERK1/2 signaling to induce shedding of exosome-size EVs, and that loss of Diaphanous-related formin-3, a cytoskeletal regulator, also promotes the release of EVs in amoeboid prostate cancer cells [141]. Recently, EGF signaling facilitating MVs biogenesis was illustrated by the study reporting that Rho family small G protein Cdc42 activated by EGF binds to IQGAP1 to promote MVs shedding and maintains EGF signaling by blocking EGFR endocytosis and then helps MVs biogenesis (positive feedback) (Fig. 3) [113]. Additionally, Rab11/11a-positive compartments were identified as novel sites of exosome biogenesis and these latter exosomes appear to be preferentially released when nutrients stress or Akt/mTORC1 inhibition occurs (Fig. 2) [142].

### TNFR regulates EV biogenesis and release

TNF (tumor necrosis factor) and TNFR (TNF receptor) superfamilies, consisting of 19 ligands and 29 receptors respectively and are associated with many inflammatory disorders [143]. It has been reported that TNF-α modulates the protein and mRNA content of exosomes derived from the endothelial cells and the small intestinal epithelial cells, suggesting inflammatory signals might regulate the biogenesis of exosomes [144, 145]. Meanwhile, some studies showed increase of exosome and microvesicle production in response to inflammatory stimuli [146–148]. For example, Sohda et al. demonstrated that TNF-α trigger the release of TNFR1 containing EVs from the human bronchial epithelial cells, which depends on acid- and neutral-sphingomyelinase [148]. It was reported that TNF influences the expression level of partial proteins in EVs and increases the rate of vesiculation in the brain endothelial cells [149]. In hepatocytes, toxic lipid mediates the release of EVs by death receptor 5 (DR5 or TNFRSF10B) proapoptotic signaling, which consists of a CHOP-DR5-caspase8-caspase3 signaling cascade activating ROCK1 (Fig. 3) [150]. Implicitly, targeting ROCK1 suggests that lipotoxicity-induced EVs are generated from the plasma membrane [150]. In addition, cell death inducers bind to TNFR and activate RIPK3, which was described to phosphorylate the pseudokinase mixed lineage kinase domain-like (MLKL) (Fig. 2) [151]. Mechanically, this phosphorylation was found to improve MLKL association with the endosomes and promote endosomal trafficking, resulting in enhanced release of EVs containing ph-MLKL in the colorectal adenocarcinoma cells [151]. It should be noted that this enhancement is not the result of necrosis. Furthermore, the release of exosomes in this pathway during necrosis, which is Rab27-independent, may prefer lysosomal exocytosis to MVB-mediated exocytosis [152].

### Wnt signals regulate EV biogenesis and release

The Wnt pathway plays key roles in short range cell–cell signaling within specific tissues, which is essential for both developmental and homeostatic processes, often divided into β-catenin dependent and independent signaling [153–155]. Interestingly, Wnts are involved in a complicated signaling network and crosstalk with the Notch, EGF, Hippo, and FGF pathways [154]. A genome-wide miRNA and CRISPR/Cas9 screen targeting Wnt signaling and trafficking-related genes identified multiple mediators of EVs secretion [121]. It was demonstrated that Wnt-mediated GSK3 inactivation downregulates Rab27 mRNA and protein regulating EVs release and that β-catenin/TCF-4 activates the expression of Rab27B, which is known to be required for the release of exosomes from colorectal cancer stem cells (Fig. 2) [121, 156]. β-catenin-independent signaling and Wnt components affect cytoskeletal rearrangements, cell motility, induced invasiveness, and factors of EVs release such as Rabs, Rho-GTPases, Ca^2+^, and ARF6 [157]. Consistently, Wnt5A, a non-canonical Wnt protein, regulates Ca^2+^–dependent EVs release in an auto- or paracrine manner in malignant melanoma cells and enhances exosome release during Wnt5a/PI3K/miR-122 mediated hepatocyte differentiation [158, 159].

### Hypoxia affects EV biogenesis and release

Oxygen is essential for energy metabolism, and hypoxia is considered as a hallmark of the tumor microenvironment due to uncontrolled proliferation of tumors [160]. Many studies have shown that hypoxia enhances the release of EVs from different cancer cells and alters cargo at the expression level [161–168]. Interestingly, hypoxia can promote human umbilical cord mesenchymal stem cells to release more EVs that exhibit enhanced anti-inflammation and anti-fibrosis potential, while hypoxia leads to larger EVs from adipose mesenchymal stem cells and smaller EVs from pancreatic tumor cells [169–171]. Such variety implies the existence of different regulation pathway in each event of EV biogenesis. In breast cancer cell lines, exposure to hypoxia significantly increases the number of exosomes in media, which may be mediated by hypoxia-inducible factor-1α (HIF-1α), a subunit of the major component of the hypoxia-related signaling pathway [162]. In ovarian cancer, exosome release increasing is facilitated by upregulating Rab27A, downregulating Rab7, LAMP-1/2, neuraminidase-1, and by promoting a more secretory lysosomal phenotype [172]. Further, the regulation of Rab proteins by hypoxia is mediated by STAT3, which indicating certain cytokines as signals also triggering STAT may affect the release of EVs [172]. Consistently, the secreted interleukin-25 from lung epithelial cells downregulates Rab27A/B expression in macrophages to suppress exosome release, and, of note, lipopolysaccharide can also stimulate marcophages to release exosomes [146]. For the balance between MVB biogenesis and degeneration, hypoxia activates the oxidized ataxia telangiectasia-mutated gene (ATM) in breast cancer-associated fibroblasts, which impairs lysosome functions by phosphorylating a subunit of proton pump in the lysosomes to enhance autophagy-associated exosome release [173]. Hypoxia increases expression and activation of some cell surface receptors through HIF, leading to induced endocytosis and exosome release [174]. There are some studies concerning hypoxia affecting the shedding and the content of microvesicles, but few mechanisms were proposed [166, 175]. It was reported that hypoxia augments microvesicle shedding in human breast cancer cells by activating HIF and regulating the expression of Rab22a that localizes to budding microvesicles [176].

### Other extracellular signals affect EV biogenesis and release

Cells are in a complicated and constantly changing environment containing multiple factors such as pH, thermal and oxidative stress, nutrients, light, and physical stress [142, 162, 177–182]. There are some other extracellular signals affect EV biogenesis, and it is noteworthy that some unconventional forms of extracellular signals can regulate EV biogenesis [183]. Circadian clock, responsible for regulating many aspect of physiology in mammals, was found to regulate the proteome of small EVs in tendon fibroblasts, and the abundance of matrix metalloproteinase 14 in small EVs is regulated by flotillin-1 [184]. Extracellular matrix (ECM) constitutes the physical component of the extracellular milieu, and some studies demonstrated that ECM stiffness and mechanical force can regulate the EV secretion [185–188]. Intriguingly, Liu et al. found that the ECM mechanical force-induced exosomes have regulated miRNAs which can transmit the mechanical force to modulate cancer metastasis [189]. Similarly, shear force is an important signal regulating microvesicle release in vascular environment [179]. This kind of signal was demonstrated necessary for platelets to expose the phospholipid phosphatidylserine on the outer membrane surfaces and release microvesicles [180]. This process depends on integrin which serves as a mechanical sensor and a signal transducer, and downstream Src/Rac1 signaling pathway [180]. Ayers et al. have reviewed the effect of shear stress, as well as other stimuli within the cardiovascular system (acute cardiac stress, hypertriglyceridaemia, inflammation, etc.), on the release of microvesicles, and proposed that the degree of shear triggering the microvesicle release from different cells is not the same [179].

**Fig. 4:**
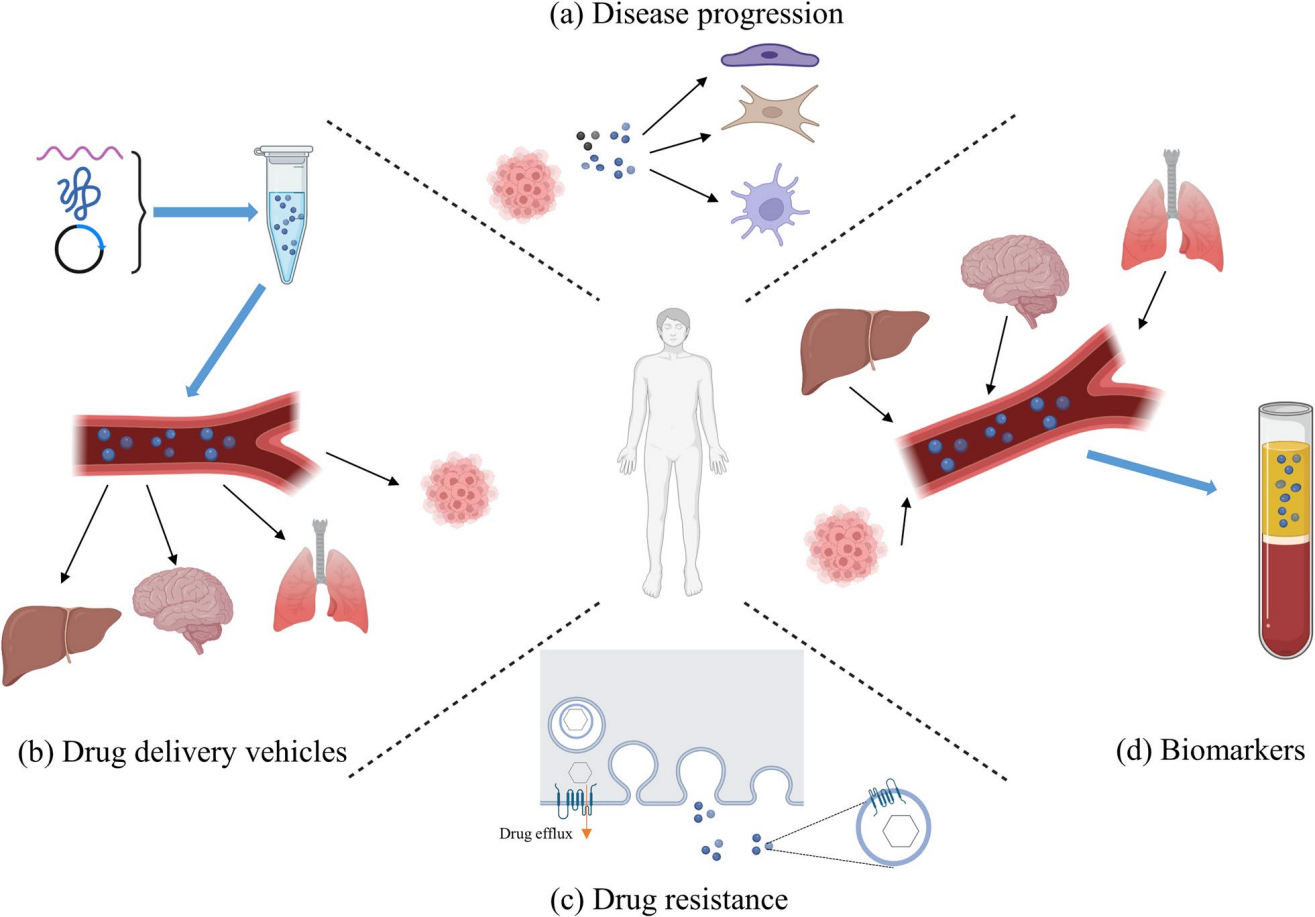
The diagram of EVs in clinical application. **a** EVs participate in various disease progression including cancer, infectious diseases and neurodegenerative disorders. For example, tumor-derived EVs can help pre-metastatic niche formation, act within tumor environment by educating different types of stroma cells and propagate tumor heterogeneity. EVs become potential novel targets for therapeutic intervention. **b** The low immunogenicity, efficiency and stability of EVs make them promising vehicles for drug delivery. Exogenous and endogenous loading approaches can be applied. For example, EVs can be loaded with specific cargo through direct transfection, and cells can be engineered to express the therapeutic of interest. In addition, EVs can also be modified to help deliver to the desired site of action. **c** EVs contribute significantly to drug resistance. Exosomes encapsulate and export drugs, horizontally transfer drug efflux pumps to recipient cells, and transfer biomolecules that promote drug inactivation. **d** EVs reflect heterogeneous biological changes related to diseases, supporting disease prediction, diagnosis, prognosis and surveillance with simplicity and stability. Created with BioRender.com

The regulatory mechanisms mentioned above are briefly illustrated in Fig. 3 and Fig. 4, which demonstrate that information flows in, is transduced through a complex but precise pathway, and an appropriate response is delivered. Moreover, it is conceivable that these pathways intersect in a complex but precise network that is regulated on several different levels.

## Clinical relevance of the biogenesis and release of extracellular vesicles

As research in the field of EVs intensifies, EVs show promise for clinical therapies. For example, EVs play important roles in disease pathogenesis, especially in cancer. In the studies described above, the aberrant environment caused by diseases provokes the abnormity in the biogenesis of EVs, such as increase of circulating metabolites, gene mutation at a high frequency and nutrient deficiency in cancer, inflammation, oxidative stress related to hypoxia, and mechanical force changes during cancer metastasis [113, 129, 142, 148, 173, 189]. More significantly, it is implied that signals transformed from extracellular environment changes regulate EV biogenesis, and in turn, these EVs further deliver information to other unaffected cells to provoke extracellular signaling cascades, and finally aggravate diseases. Many studies have shown that EVs drive the formation of a pre-metastatic tumor niche and stimulate tumor progression via inducing proliferation, facilitating angiogenesis, and promoting immune escape (Fig. 4) [14]. In this context, strategies that inhibit the biogenesis and release of EVs are potent to treat EVs-driven diseases [14]. In addition, there is growing evidence that biomolecules in EVs can be used as biomarkers for early detection and monitoring of diseases involving the central nervous system, liver, kidney, lung, etc. (Fig. 4) [190]. Further, EVs have many characteristics, such as intrinsic stability and inherent targeting properties, that make them promising drug delivery vehicles (Fig. 4) [191].

However, with increasing expectations for the application of EVs, the mechanisms regulating the biogenesis and release of EVs have become an inevitable question. For example, the heterogeneity of EV content from different cells challenges the drug carrier development, though it is the foundation of its application to liquid biopsy. Why cells produce EVs and whether external perturbations modulate the extent of EV biogenesis, processing, and release of EVs are still unknown. Significantly, studies on the regulation by extracellular signals can be of value in directly improving the efficiency of cargo loading and EVs release in drug delivery, effectively inhibiting pathogenesis, restraining EVs-mediated drug resistance, comprehensively assessing cancer diagnosis, prognosis, and progression, and screening for therapeutic drug-induced cellular stress [14, 191–194].

In recent years, technologies for EV application have been refining. As an example, exosomes were engineered to carry short interfering RNA or short hairpin RNA specific to oncogenic KrasG12D, and treatment with these exosomes suppressed cancer in mouse model of pancreatic cancer [195]. For such drug delivery and cell-free cell therapy, EV production, isolation, purification and drug loading are general processes. The drug loading includes endogenous methods, which equip vesicles with drug or biomolecules in parent cells using nanoparticles or genetic engineering, and exogenous methods, which load drugs into isolated EVs through incubation, attachment, chemical modification (e.g. click chemistry) and permeabilization (sonication, electroporation and saponin treatment) [196–198]. For EV production, to produce sufficient numbers of EVs, introducing external stimuli to regulate EV release is more applicable than genetic methods since the natural secretion process is required in some cases [181]. For example, phototherapy-based LED light was applied to increase the dendritic cell-derived EVs that have been developed into clinical trials, and more importantly, the immunomodulation function, immunogenicity, oxidative stress levels, and biocompatibility of them are comparable to naturally released EVs [181]. A tubular perfusion bioreactor culture system with 3D-printed scaffolds was applied to enhance EV production, in which appropriate flow rate and produced shear stress leaded to more than 100-fold increase in endothelial cell EV production than 2D flask culture [199]. The addition of chemicals to preparing system to boost EV production has been reported. Using sodium iodoacetate and 2,4-dinitrophenol to inhibit glycolysis and oxidative phosphorylation can trigger a three- to twenty-four- fold increase in the secretion of EVs with similar property and function as those from untreated cells [200]. In practice, many methods have been considered to solve the problem of EV application, not only how to produce EVs on a large scale. However, manipulation of cargo abundance and signaling pathways using external signals should be cautious, and evaluation of the property and efficacy of produced EVs is essential to avoid risks.

## Conclusions

Since EVs were first described to deliver mRNAs and microRNAs between cells in 2007, much advances and insights have been made in the biogenesis, release, and functions of EVs, as we discussed above [24]. As an intracellular-derived vesicular structure, the biogenesis process of EVs is consistent with the intercellular vesicles transport system, such as the pattern of cargo sorting, trafficking, and fusion with the target membrane have been described. However, studies on the biogenesis and release of EVs is just beginning and the specific molecule mechanisms remain unexplored.

A single cell hosts different EVs subpopulations with different functions. EVs subpopulations and functions depend on their cargo, which in turn depends on the sorting machineries and signaling regulation. While this implies that distinct mechanisms may act simultaneously or sequentially on exosomes or microvesicles, the precise regulatory mechanisms need to be further explored. Specifically, the sorting process of certain cargo such as miRNAs, the correlation between cargo and machineries, the establishment of the balance between degradation and secretion of MVBs, and the crosstalk between different mechanisms are unclear.

Studies reported that some components of classical signaling pathways were found to be involved in the biogenesis and release of EVs, which implies their necessity. Indeed, extracellular signals can regulate the biogenesis and release of EVs mainly by binding receptors and activating cascades. Major regulatory effectors are Rabs, Rhos (especially Cdc42), Ca^2+^ and corresponding events are the transport of MVBs and cytoskeleton rearrangement, but other effectors regulated by extracellular signals remain to be revealed. Interestingly, the cellular signaling pathways comprise an intricate network where a single molecule can be activated by several upstream signals and can activate different effectors. It remains to be elucidated whether a single extracellular signal regulates multiple EVs subpopulations biogenesis and release, or whether a process is regulated by multiple signals. Therefore, other relevant signaling pathways should be taken into account in the study of this issue.

It should be noted that several signaling cascades have been identified that utilize EVs for signaling transport in the tumor-stroma interaction, including potentially oncogenic signaling cascades and signaling cascades associated with tumor progression and metastasis, such as Wnt, TGF-β, PD-L1, EGFR [110, 155, 157]. Even mechanical force signals can be transmitted by exosomes [189]. In this case, EVs with signaling are themselves a form of extracellular signaling that allows signaling to transfer at the intercellular level. Thus, extracellular signaling likely promotes the biogenesis of EVs carrying components of signal cascade, which in turn promotes the biogenesis of EVs and play other roles in recipient cells.

In conclusion, there are good reasons to expect that EVs will make great contributions to all biological events. EVs are not only responsible for mediating information transfer between different cells, but also extend the concept of signal transduction to intercellular aspect by working as the collection of information with extraordinary efficiency, specificity and stability. Despite the growth of therapeutic values of EVs, translating of EVs into clinical applications remains a challenge. Understanding of regulation of biogenesis and release by extracellular signals is necessary for supporting optimal utility and determines the potential of applications.

## Data Availability

Not applicable.

## Abbreviations

ALIX: ALG-2 interacting protein X
ARRDC1: Arrestin domain-containing protein 1
ATM: Ataxia telangiectasia-mutated gene
CHMP2-4: Charged MVB protein 2-4
DAG: Diacylglycerol
DR5: Death receptor 5
DRF3: Diaphanous-related formin-3
ECM: Extracellular matrix
EGFR: Epidermal growth factor receptor
ERK: Extracellular signal regulated kinase
ESCRT: Endosomal Sorting Complex Required for Transport
EV: Extracellular vesicle
GAP: GTPase activating protein
GDI: GDP dissociation inhibitor
GEF: Guanine nucleotide exchange factor
GPCR: G protein-coupled receptor
HIF-1α: Hypoxia-inducible factor-1α
hnRNPA2B1: Heterogeneous nuclear ribonucleoprotein A2B1
HRS: Hepatocyte growth factor-regulated tyrosine kinase substrate
ILV: Intraluminal vesicle
ISEV: International Society for Extracellular Vesicles
miRISC: MiRNA induced silencing complex
MLKL: Mixed lineage kinase domain-like
MV: Microvesicle
MVB: Multivesicular body
nSMase2: Neural sphingomyelinase 2
PKC: Protein kinase C
PTM: Post-translational modification
ROCK: Rho-associated protein kinase
RTK: Receptor tyrosine kinase
Shp2: Src homology 2-containing protein tyrosine phosphatase 2
SNARE: Soluble NSF-attachment protein receptor
STAM: Signal transducing adaptor molecule
TFR: Transferrin receptor
TNF: Tumor necrosis factor
TNFR: TNF receptor
VAMP7: Vesicle-associated membrane protein 7
